# Off-pump Myocardial Revascularization — From the Beginning Till Now

**DOI:** 10.21470/1678-9741-2024-0993

**Published:** 2025-02-05

**Authors:** Enio Buffolo, Tomas A Salerno, Ricardo C. Lima

**Affiliations:** 1 Division of Cardiovascular Surgery, Escola Paulista de Medicina, Universidade Federal de São Paulo, São Paulo, São Paulo, Brazil; 2 Division of Cardiovascular and Thoracic Surgery, Jackson Memorial Hospital, University of Miami, Miami, United States of America; 3 Division of Thoracic Surgery, Universidade de Pernambuco, Recife, Pernambuco, Brazil

Surgical myocardial revascularization debuted in the cardiovascular surgical practice late, just after two decades of experience in congenital heart disease and valve dysfunctions.

The landmark of direct myocardial revascularization was the possibility to access the coronary arteries through coronary angiography developed by Mason Sones in the Cleveland Clinic, in the early 1960s^[1]^.

The identification of the presence and severity of coronary obstructions was the birth of modern coronary surgery and, more than this, it allowed the evaluation and selection of really effective surgical technics after many ineffective indirect surgical procedures made in the past.

The modern era of direct myocardial revascularization started with the saphenous bypass vein graft^[2]^. Reports coming mainly from Cleveland Clinic, like diagnosis, selection of patients, and technical challenges, were published and made the coronary bypass graft the most common cardiac surgical procedure in the next decades.

Garrett, in 1962, utilized a saphenous bypass graft instead an endarterectomy in a patient, immediately after a conventional valve replacement due to an accident with the left anterior descending (LAD) coronary artery. He published the patency of the graft in 1973^[3]^.

Although coronary arteries are located on the surface of the heart and it is not necessary to open cardiac chambers, surgeons started to use cardiopulmonary bypass to make graft anastomoses due to the development of myocardial protection methods, familiarity with extracorporeal circuits, and mainly to perform a delicate anastomosis in an arrested heart.

The possibility to graft a beating heart, nevertheless, was applied before. Goetz et al. did a direct myocardial revascularization utilizing the mammary artery and a mechanical suture with a tantalum ring^[4]^.

Vasili Kolesov and Potashow published in Russia (1965)^[5]^, and later in the United States of America (1967)^[6]^, their experience with mammary artery grafted to LAD in a beating heart through a left thoracotomy and in a few cases with mechanical suture. It is interesting to observe that at that time they didn’t have information about the coronary arteries and made the operation only in clinical bases. Years later, Trapp & Bysaria, in Canada^[7]^, and in the same year Ankeney, in Cleveland^[8]^, independently reported the first series of patients with acceptable results. This alternative of myocardial revascularization did not have general acceptance due to technical difficulties, and the concept was that it was not possible to occlude a coronary artery, even for a few minutes, without causing myocardial infarction. Due to this concept, they applied a complicated distal perfusion device that caused difficulties to perform the anastomosis.

Only years later, Buffolo et al.^[9,10]^ and, independently, Benetti^[11]^ published a consecutive series of patients operated with saphenous or mammary arteries as grafts treating LAD and right coronary or diagonal branches calling attention to feasibility and safety of this alternative of myocardial revascularization.

In the same decade, Archer^[12]^, Laborde^[13]^, and others related isolated cases in a short experience.

Technical maneuvers were described to facilitate anastomosis as position of the table, anesthesia expertise, and pharmacological “stabilizers” like verapamil or beta-blockers to reduce hearth rate, arterial pressure, and oxygen consumption^[14]^.

Trying to avoid an incomplete myocardial revascularization, Lima applied stitches in the posterior pericardium (Lima stitch) with exposition to the posterior coronary branches to perform a complete myocardial revascularization, and this maneuver was essential to expand the indication to almost all patients^[15]^.

To facilitate the anastomoses in a quiet field, stabilizers were developed by compression or like the OCTOPUS® making a regional “cardiac arrest”. It can be used with devices like STARFISH® that put the “apex cordis” up to permit visibility of the marginal branches of circumflex artery using the described principles of the Lima stitch^[15,16,17,18]^.

The use of stabilizers was very important to get better quality anastomoses, and now they are essential in off-pumps coronary surgery.

During the next years, some papers tried to demonstrate the advances of myocardial revascularization in a beating heart without cardiopulmonary bypass but did not had wide acceptance by local or international community^[19,20]^.

Despite the evidence of the feasibility with good results, the comfort to perform a conventional coronary artery bypass in an arrested heart and the concerns about the quality of the anastomoses led to only isolated centers to adopt this alternative of myocardial revascularization^[21,22,23,24,25,26,27,28,29,30]^.

The development and persistence in performing off-pump coronary operation by pioneer groups culminated with the concept proposed by Benetti to make a mammary artery anastomosis in a beating heart through a mini left thoracotomy. His idea was presented in a meeting in Rome (1994) and received the acronym of MIDCAB (for minimally invasive direct coronary artery bypass). The clinical experience was disseminated all around the world by Benetti and Calafiore among others with the name of “LAST operation” (for left anterior small thoracotomy).^[31,32]^.

This idea was strongly attractive and was the key to return the interest in beating heart surgery. Many groups started to learn about how to operate without pump and that was really possible to achieve good quality anastomoses, even more, utilizing sternotomy.

Curiously, in 1982, we collected in our former publication only five direct reports in off-pump coronary surgery, 18 in 1992, and in 1998, an explosion of 18.423 papers in the subject.

With the widespread application of beating coronary artery surgery, many important contributions were made like the concept of hybrid approach and the use of stabilizers.

In the hybrid approach, we use a mammary artery-LAD artery anastomosis with minimal left thoracotomy and, before or after, percutaneous angioplasty to other coronary arteries combining the five-star treatment of LAD with a minimal invasive complete coronary artery disease^[33,34,35,36]^.

In the following decades, we can observe a lot of contributions and randomized controlled trials comparing off-pump *vs.* on-pump procedures regarding mortality, morbidity, inflammatory response, patency rates, stroke, blood transfusion, costs, and results in a high number of patients^[37,38,39,40,41,42,43,44,45,46,47,47]^.

Advances and criticisms by opinion makers like Thomas Salerno, Michael Mack, Gianni Angellini, John Puskas, David Taggart, Antonio Calafiore, and R. Ascione among others stablished the main concepts of this alternative of myocardial revascularization. The different results regarding patency rates and benefits and risks in our opinion are mainly due to improper training and selection of patients.

Although some groups are now performing 90% or even 100% of cases, we believe that it is difficult to achieve a full to application of the technique. Patients with severe or multiple lesions, with previous stenting, very hypertrophic ventricles, and intramyocardial arteries among others need the conventional approach, and this is the key to avoid unacceptable conversion rates that increases mortality. It is also important to the anesthetist

to be familiar with the procedure because he/she is part of the success providing pressure control and avoiding high heart rates. In a retrospective analysis of our experience, we think that offpump myocardial revascularization have strong evidences of advantages regarding reduction in mortality rates, reduction in strokes, lower major postoperative complications, short hospital stay, and lower costs. Perhaps it will be necessary in a near future to justify a sub-specialty in coronary surgery, offering to the patients the two options of surgical myocardial revascularization and the application of the new technology.

The technique using port-access and robotics represents the maximum application of a minimally invasive concept using the technology of the new millennium, and the applicability and long-term results will be necessary to justify an increased use of this alternative.

In low-risk patients, it is difficult to detect advantages of off-pump *vs.* on-pump procedures due to the good results of the standard revascularization with cardiopulmonary bypass and cardioplegia. The differences appear in high-risk patients — “Worse the patients, better the outcome”.

Comparative studies between off-pump coronary surgery and angioplasties will be necessary to stablish new strategies in the management of coronary disease because the parameters we have now compare conventional surgery with percutaneous intervention in the majority of controlled trials.

Finally, myocardial revascularization without cardiopulmonary bypass is a five-star treatment of coronary insufficiency with applicability varying from surgeon to surgeon, being essential a training period to avoid the learning curve.
